# When the Algorithm Becomes the Dietitian: Large Language Model Hallucinations, Algorithmic Food Environments, and the Erosion of Nutritional Autonomy

**DOI:** 10.3390/nu18193128

**Published:** 2026-09-23

**Authors:** Ismail Dergaa, Rym Ben Othman, Hatem Ghouili, Halil İbrahim Ceylan, Nizar Souissi, Souhail Bchini, Valentina Stefanica, Raul Ioan Muntean, Nicola Luigi Bragazzi

**Affiliations:** 1 High Institute of Sport and Physical Education of Ksar Said, University of Manouba, Manouba 2010, Tunisia; phd.dergaa@gmail.com (I.D.); n_souissi@yahoo.fr (N.S.); 2 Research Unit Physical Activity, Sport, and Health, UR18JS01, National Observatory of Sport, Tunis 1003, Tunisia; 3 Institut National de Nutrition et de Technologie Alimentaire de Tunis, 11 Rue Jbal Lakhdhar, Tunis 1007, Tunisia; benothmanr@gmail.com; 4 Faculty of Medicine of Tunis, University of Tunis El Manar, Tunis 1007, Tunisia; 5 Research Unit Sport Sciences, Health and Movement (UR22JS01), High Institute of Sport and Physical Education of Kef, University of Jendouba, Kef 7100, Tunisia; hatemghouili@gmail.com (H.G.); souhail.bchini@gmail.com (S.B.); 6 Physical Education and Sports Teaching Department, Faculty of Sports Sciences, Ataturk University, Erzurum 25240, Türkiye; 7 Department of Physical Education and Sport, Faculty of Sciences, Physical Education and Informatics, National University of Science and Technology Politehnica Bucharest, Pitesti University Center, 110040 Pitesti, Romania; valentina.stefanica@upb.ro; 8 Faculty of Sciences, Department of Physical Education and Sport, “1 Decembrie 1918” University of Alba Iulia, 510009 Alba Iulia, Romania; 9 Department of Clinical Pharmacy, Saarland University, 66123 Saarbrücken, Germany; nicola.bragazzi@uni-saarland.de

**Keywords:** large language models, hallucinations, dietary behavior, nutritional misinformation, social media, recommender systems, food autonomy, digital health, behavioral economics, ADRIFT

## Abstract

Large language models (LLMs) can answer dietary questions, but their use as substitutes for registered dietitians remains insufficiently quantified. Evaluations have identified inaccurate nutrient targets, incomplete clinical advice, and insufficient personalization, alongside useful performance on some general nutrition tasks. Social media and food-delivery interfaces may also influence dietary choices through food cues, social endorsement, and choice architecture. This conceptual perspective presents ADRIFT (Algorithmic Dietary Reconfiguration through Informational, Framing, and Technological mechanisms) to examine how these influences might interact. Drawing on a targeted, nonsystematic synthesis of nutrition, behavioral, neuroscience, and AI literature, ADRIFT distinguishes three axes: informational errors and unsupported advice; framing through content and interface design; and adaptive personalization and cognitive delegation. Direct evidence supports selected component mechanisms, but their convergence into a feedback loop that reduces nutritional autonomy remains hypothetical. We propose a six-category taxonomy covering hallucinations and related nutrition errors, define nutritional autonomy erosion as declining capacity for informed, self-directed dietary decisions, and outline eight testable hypotheses. The framework concerns unsupervised use and does not predict that language models will replace dietetic professionals. Longitudinal validation, platform experiments, and clinical safety evaluations are needed to determine whether, for whom, and under which conditions the proposed harms occur.

## 1. Introduction

Digital platforms mediate access to food, exposure to dietary messages, and opportunities to seek nutritional guidance. Social media and recommender systems can shape the information and choices users encounter, while generative artificial intelligence (AI) can produce dietary explanations and meal suggestions [1,2,3,4,5,6]. Their influence depends on the platform, its objectives, and the context of use. Commercial incentives may conflict with nutritional goals, but this conflict should be demonstrated for specific systems rather than assumed to characterize all dietary AI.

Three developments motivate the present analysis. Social media brings food imagery, influencer advice, and commercial promotion into everyday information streams [1,2]. Food-delivery interfaces can alter the effort required to compare and order meals, while conversational recommender systems can influence how options are presented [3,4]. General-purpose LLMs add fluent responses to questions about food, weight management, supplements, and chronic disease [5,6]. These functions may be useful, but persuasive presentation and accurate clinical guidance are separate properties.

The prevalence of dietary LLM use and its substitution for professional consultation require direct measurement. Evaluations of generated advice establish capabilities and limitations, not the number of people relying on that advice. Individuals with type 2 diabetes, chronic kidney disease, food allergy, or complex medication regimens may require guidance that depends on disease stage, treatment, laboratory results, and individual circumstances. Existing nutrition evaluations do not establish the safety of unsupervised general-purpose LLM use for such decisions [7,8]. Clinical rules, verified relevant information, and professional oversight are safeguards to evaluate, rather than guarantees of reliability.

Social media may compound these concerns through commercial food promotion and perceived influencer authority [1,9,10,11]. Experimental and neuroimaging studies provide evidence that food cues affect attention, reward-related responses, and some eating outcomes [12,13,14,15]. We hypothesize that personalized content and ordering interfaces may amplify or modify these effects [3,4]. ADRIFT addresses the possible interaction of these channels; it does not assume that an integrated population-level feedback loop has been demonstrated.

This perspective addresses three linked questions. How can hallucinations and other LLM nutrition errors be organized into clinically useful evaluation categories? How should nutritional autonomy be distinguished from knowledge, self-efficacy, and diet quality? Under what conditions could information errors, food-content framing, and adaptive recommendation interact? General hallucination research provides a foundation [16,17], while accounts of cognitive delegation suggest adjacent theoretical concerns [18]. We develop a nutrition-specific synthesis without claiming that these questions or their individual mechanisms have not previously been studied.

We first examine dietary LLM use, social-media influence, and clinically relevant error patterns. We then consider choice architecture, food-cue reward processing, and possible interactions across services. The ADRIFT framework integrates these mechanisms, distinguishes empirical findings from extrapolation, and defines candidate measures of nutritional autonomy erosion. Eight hypotheses in Section 12 specify how the model could be tested. The purpose is to identify conditions under which AI may undermine or support informed dietary choice and to guide proportionate clinical and governance responses.

This article is a conceptual perspective, not a systematic or scoping review. Supporting literature was identified through targeted, nonsystematic searches of PubMed/MEDLINE, Scopus, and Web of Science using combinations of terms relating to LLMs, dietary behavior, social media, recommender systems, ultra-processed foods, and reward processing. Sources were selected for conceptual relevance, prioritizing primary studies and relevant reviews. We did not preregister a protocol, conduct duplicate screening, apply a formal risk-of-bias instrument, or produce a Preferred Reporting Items for Systematic Reviews and Meta-Analyses (PRISMA) flow diagram. The six-category taxonomy groups reported or clinically plausible error patterns; it was not derived from a systematically coded corpus or validated by an expert-consensus procedure. Narrative synthesis and concept analysis organized the ADRIFT axes around information content, choice framing, and adaptive technology. The synthesis cannot establish completeness, comparative effect sizes, or causal relationships between the axes. The framework concerns general-purpose generative LLMs and consumer-facing recommender and food-delivery interfaces. It does not address knowledge-based or rule-based clinical decision support systems, nor validated task-specific machine-learning tools such as nutritional screening models, which follow different development, validation, and regulatory pathways and should not be assumed to carry the same risks.

## 2. LLMs as Dietary Advisers and the Epistemic Illusion

Evaluations suggest that LLMs can answer some general nutrition questions adequately, while performance varies with the model, prompt, clinical scenario, and assessment criterion [5,7,8,19,20]. These studies primarily assess outputs or hypothetical cases; their results should not be equated with clinical effectiveness or safety in routine use.

Waiting times, cost, geography, and limited access to dietitians may encourage people to seek readily available automated advice. LLMs provide immediate, conversational responses, and their performance on nutrition-knowledge tasks can appear strong compared with that of human test takers [5]. The proposed safety concern arises when users infer individualized clinical competence from this performance or from the style of a response.

We use epistemic illusion to describe a proposed mismatch between perceived competence and independently assessed advice quality. An LLM may produce a plausible meal plan without verified information about renal function, medications, intake, or treatment goals. Comparisons with clinical or dietitian standards have identified limitations in personalization and nutritional adequacy [7,8,20]. In a separate study of online medical questions, evaluators rated chatbot responses favorably for quality and empathy [21]. That finding concerns evaluator judgments; it does not establish patient trust, adherence, or clinical benefit. Whether presentation causes users to overestimate dietary competence remains an empirical question.

Evaluations of AI-generated exercise prescriptions similarly show why expert review may be needed before implementation [22,23]. The AI-Chatbot Induced Cognitive Atrophy (AICICA) account proposes that habitual delegation could weaken practiced cognitive skills [18]. We treat this as an adjacent conceptual argument, not evidence that dietary AI causes cognitive decline.

## 3. Social Media and Perceived Dietary Authority

Social media gives both qualified professionals and non-credentialed creators opportunities to influence dietary beliefs. A systematic review covering 96 studies reported associations between food and nonalcoholic beverage marketing and increased intake, choice, and preference in children and adolescents [14]. Its evidence base spans marketing formats; it does not isolate the additional effects of AI personalization. The relevant mechanisms include social endorsement, influencer identification, and the salience of visual food cues.

Likes, comments, and shares may act as social endorsements of dietary content, irrespective of its nutritional quality [1]. In a cross-sectional study of adult followers, Shabahang et al. found associations between parasocial attachment to a favorite food influencer and measures of disordered eating, food addiction, and grazing [10]. The design cannot determine causal direction: existing eating difficulties may also influence attachment and content selection. These findings support investigation of perceived dietary authority, without establishing that influencer attachment causes eating disorders.

Food-advertising and food-cue studies report responses in brain regions involved in valuation, motivation, and sensory processing [12,13,15]. Such findings provide a plausible basis for studying cue-driven food choice. However, blood-oxygen-level-dependent signals from functional magnetic resonance imaging (fMRI) do not directly measure dopamine release, and regional activation alone does not establish addiction, impaired self-control, or subsequent consumption. Evidence from controlled stimuli also cannot establish the effects of prolonged exposure to a personalized social-media feed.

Recommendation systems may amplify food content that elicits engagement, including commercially promoted or misleading messages [1,9]. A scoping review documented food and beverage advertising on social media and raised concerns about the promotion of nutritionally poor products, including exposure among younger users [24]. The relative reach of accurate and inaccurate dietary content, and the contribution of algorithmic ranking to that reach, require direct audits. Neither misinformation research nor advertising prevalence alone establishes that every platform favors nutritionally inaccurate advice.

## 4. Hallucinations and Related Nutrition Errors

Hallucination refers here to generated content that is fabricated, factually incorrect, or unsupported by the relevant source or context [16,17,25]. Confident wording may obscure such errors, but confidence is not a defining feature. Incomplete advice, arithmetic errors, inadequate personalization, and representational bias may create clinical risks without being hallucinations in the strict sense. Table 1 therefore presents a proposed six-category taxonomy of hallucinations and related nutrition errors. Its categories can overlap, and its qualitative risk levels describe potential consequences rather than measured incidence or a validated severity scale. The six categories describe error patterns identified in the retrieved literature and in clinical reasoning; they are not an exhaustive enumeration of possible LLM nutrition errors, and further categories may emerge as evaluation methods develop. The entries in Table 1 are illustrative constructions intended to support evaluation design, not documented outputs of any named system or validated clinical cases.

Drug–nutrient interaction omissions deserve attention because an otherwise plausible diet may be unsafe with a particular medication. Grapefruit can inhibit intestinal CYP3A4 and alter exposure to some statins, calcium-channel blockers, and immunosuppressants; this does not apply uniformly to every drug in those classes [27]. This is an established pharmacological example for testing LLM advice, not a quantified hallucination pattern demonstrated by general AI commentaries [17,25]. Condition-specific evaluations also illustrate the limits of generic advice. Studies of personalized dietary prescriptions and chronic disease scenarios identify shortcomings in nutritional adequacy and individualization [8,20], while evaluation of food-allergy diets identified potentially unsafe recommendations [28]. Such findings concern generated outputs; they do not provide rates of patient injury.

Insufficient personalization is distinct from an isolated factual mistake: a response may be broadly correct yet inappropriate for the person receiving it. Liao et al. assessed scenario responses using ratings from 30 dietitians and separately administered an objective nutrition-literacy test [26]. The proportions of scenario-level indicator ratings judged fully achieved ranged from 7.50% to 37.56%; the separate objective test accuracy was 84.38%. The lower range is therefore not an overall factual-accuracy score. The study illustrates the difference between answering knowledge questions and providing complete, practical guidance, but does not itself measure user overconfidence or autonomy loss.

## 5. Choice Architecture in Digital Food Environments

Food choice responds to how options are presented, ordered, framed, and contextualized [29]. Digital interfaces can implement these design choices repeatedly and adapt them to user behavior. Persuasive technology and conversational food recommenders illustrate possible mechanisms, including those intended to support healthier choices [3,4]. Their effects depend on the design objective and implementation.

Commercial food-delivery interfaces may prioritize conversion, engagement, or revenue, potentially through sponsored placement, default sorting, add-ons, or salient promotional cues. The persuasive-design literature supplies candidate mechanisms [3,4], but does not establish their prevalence or nutritional effects across commercial platforms. Platform audits should document which features are present, and randomized interface experiments should test their effects while holding menus and prices constant.

A persuasive feature should not automatically be classified as a dark pattern. The concern is whether a design deceives, obstructs, or pressures users, for example through fabricated popularity claims or concealment of relevant information. Less prominent nutrition information or personalized sorting may warrant scrutiny, but their classification requires evidence about implementation and user understanding. Audits should distinguish ordinary merchandising, transparent nudges, and designs that impair informed choice.

The ultra-processed-food literature provides relevant downstream context. In a controlled inpatient trial, participants offered an ultra-processed diet consumed approximately 500 kcal more per day than when offered an unprocessed diet matched on several presented characteristics [30]. This finding establishes a consequence of dietary exposure, not a causal effect of delivery algorithms. ADRIFT therefore advances a testable hypothesis: if commercial interfaces disproportionately rank or reinforce ultra-processed options, they may increase their selection. Platform-level audits and randomized matched-menu studies are needed to evaluate this pathway.

## 6. Food Cue Reward Processing and Algorithmic Personalization

Dopamine contributes to incentive salience, learning, and motivated behavior; it should not be treated as a direct measure of pleasure or nutritional value [31]. Food choices arise from interactions among reward-related processing, physiological signals, learned preferences, and cognitive goals. Food-cue and marketing studies provide evidence relevant to some of these processes [12,13,15], but do not demonstrate that recommender systems directly target a measurable dopamine profile.

The ultra-processed-food literature provides a complementary basis for hypotheses about intake. The NOVA classification concerns processing, and is not interchangeable with a measure of palatability or addiction [32]. Hall et al. demonstrated greater energy intake under an ultra-processed dietary condition [30]; the trial did not establish that all such products override satiety through a single dopamine mechanism. Reviews discuss possible reward-related and other contributors to overconsumption [33,34]. The proposed algorithmic hedonic feedback loop in Figure 1 connects these ideas to personalization at a behavioral level. It does not represent a demonstrated neurobiological pathway.

Recommendation systems can use behavioral signals such as clicks and ordering history to adapt what users see. We hypothesize that repeated personalization may reinforce familiar choices and increase exposure to cues with learned motivational salience. Studies of food valuation and self-control provide a rationale for examining interactions with user goals [35], but do not establish that personalization blunts satiety, causes addiction, or changes prefrontal function. These proposed consequences require longitudinal and experimental testing, including comparisons with personalization designed to support users’ stated dietary goals.

## 7. Potential Convergence Across Digital Food Services

LLM functions, social-media content, food-delivery interfaces, and wellness applications may be encountered within the same person’s daily routine. Coordinated exposure could therefore occur even without a single integrated platform. ADRIFT treats their convergence as a plausible configuration requiring platform-specific and user-level investigation (Figure 2). Evidence about chatbot response quality [21] does not, by itself, demonstrate integration or cross-service data sharing.

A relevant scenario is conversational dietary guidance embedded in a commercial ordering interface. If the assistant’s recommendations are optimized for sales, and those incentives are insufficiently disclosed, advice may conflict with the user’s health goals. This conditional scenario should be assessed through platform audits rather than assumed to describe all current deployments. The key questions are which objective governs recommendations, whether users understand it, and whether clinical or nutritional safeguards constrain it.

A second potential convergence mechanism operates through data infrastructure. Ordering history, LLM query patterns, social-media engagement, and fitness-application data can contribute to behavioral profiles when systems are technically and legally permitted to combine or infer them. Such integration could enable more precise targeting of dietary content than any one channel alone. The extent to which this occurs in practice requires platform-specific empirical investigation.

A further possibility is circular attribution: influencers and LLM outputs may repeat the same unsupported dietary claim, making repetition appear to be independent corroboration. Misinformation research motivates investigation of this mechanism [9], but the proposed cross-channel loop requires direct evidence. Children and adolescents warrant particular attention because marketing can influence food intake and preference [14]. Reviews also link social-media exposure with dietary behaviors, while emphasizing heterogeneity and uncertainty about causal direction [2,36]. Their findings do not quantify the additional effect of combined social-media and LLM use.

## 8. Cognitive Erosion of Dietary Autonomy

Dietary autonomy is the capacity to make informed, self-directed food choices that reflect personal values, relevant nutritional knowledge, and bodily experience within real-world constraints. An autonomous choice need not maximize nutritional quality. Cost, food availability, disability, culture, and household responsibilities may constrain choice without indicating cognitive impairment. Voluntary use of reliable assistance can also support autonomy when users retain the ability to understand, question, and revise recommendations.

ADRIFT proposes that repeated reliance on advice that users cannot adequately evaluate could reduce opportunities to practice independent nutritional reasoning. Nutrition evaluations show why external verification may be needed [8,20,26,28]; they do not demonstrate declining reasoning skills or interoceptive function in users. Errors may also prompt skepticism and renewed professional consultation. The adjacent AICICA account [18] remains a conceptual analogy, and both beneficial learning and harmful dependence should be considered as competing longitudinal outcomes.

Nutritional self-efficacy concerns confidence in carrying out intended dietary behavior [37]. It is distinct from knowledge and demonstrated competence: confidence can improve while performance remains unchanged, or decline as users recognize previous mistakes. Research links eHealth literacy with health behaviors [38,39], but these associations do not establish that AI delegation causes loss of self-efficacy or that literacy interventions improve diet. Repeated assessments should examine confidence, decision performance, and contextual constraints separately.

Low-friction ordering may reduce opportunities to pause and consider hunger or satiety, but it may also improve access to food and reduce practical burdens. The proposed adverse pathway is a testable interaction between interface features and eating behavior [3,4]. It should be evaluated using observed ordering patterns, repeated hunger and satiety ratings, and controlled tasks, rather than inferred from one-tap ordering alone.

## 9. The ADRIFT Framework

ADRIFT (Algorithmic Dietary Reconfiguration through Informational, Framing, and Technological mechanisms) organizes candidate influences into three axes and a proposed outcome, nutritional autonomy erosion (Figure 3; Table 2). The axes are analytic distinctions rather than mutually exclusive technologies: a conversational recommender may affect information, framing, and adaptation simultaneously.

The Informational Axis (I) concerns inaccurate, misleading, insufficiently individualized, or biased dietary advice. Table 1 organizes candidate error patterns. The direct proposed pathway is action on clinically unsuitable advice. An indirect pathway is misplaced confidence that reduces appropriate verification or professional consultation. Output-level errors have been observed, but the frequency of these downstream behaviors and harms requires direct measurement.

The Framing Axis (F) concerns how food content and interface design affect attention, perceived norms, and the salience of options. Influencer endorsement, food imagery, rankings, and defaults may influence choices even when the underlying information is accurate. Some effects may occur with limited conscious deliberation, but susceptibility and awareness vary. Nutritional literacy and transparent interface design should be evaluated as potentially complementary safeguards.

The Technological Axis (T) concerns adaptive personalization and repeated delegation over time. We hypothesize that feedback from prior engagement may reinforce exposure patterns and change opportunities to practice dietary decision-making. Lasting changes in reasoning, self-efficacy, or responses to bodily signals are proposed outcomes requiring evidence. This axis does not assume that current systems directly manipulate dopamine or inevitably induce cognitive decline.

Evidence differs both between and within the axes. Nutrition evaluations directly support the existence of some Informational-axis errors [7,8,20,26,28], while their incidence in everyday use and effects on clinical outcomes or autonomy are uncertain. The framing axis draws on empirical marketing and food-cue findings [14,15], with extrapolation required for specific AI-driven interfaces. The technological axis is the most hypothetical: reward and valuation research [31,35] does not establish that prolonged personalization erodes autonomy. The integrated feedback pathways have not been validated as a causal model.

The model predicts interactions that future studies could confirm or refute. Social endorsement may increase acceptance of inaccurate advice; adaptive ranking may reinforce choices initially influenced by framing; and declining capacity to evaluate advice may increase subsequent reliance. These relationships need not be unidirectional. Better information, supportive personalization, or increased user expertise could interrupt or reverse the proposed pathways.

Nutritional autonomy erosion denotes a within-person decline over time in the capacity for informed, self-directed dietary decisions. We use dietary autonomy and nutritional autonomy for the same domain-specific capacity. Nutritional autonomy collapse denotes a proposed severe endpoint involving sustained difficulty evaluating or revising advice and making decisions without unwanted dependence. It is not a diagnosis, and no validated cutoff is available. High use, voluntary delegation, low self-efficacy, or poor diet quality alone does not establish erosion. Unlike domain-general cognitive-delegation accounts [18], this construct concerns food-related decisions and requires validation in that context.

Operationalization should keep exposure, mechanisms, and outcomes distinct. Exposure measures include use frequency and the proportion of decisions for which advice is sought or accepted; these are not impairment measures. Candidate autonomy outcomes include unaided identification of unsafe advice, justification of food choices in relation to personal goals, and ability to reject or revise an unsuitable recommendation. Diet quality should be assessed separately. A preregistered longitudinal model may test self-efficacy or responses to hunger and satiety as mediators, but should not simultaneously use the same measure to define the outcome and claim mediation without an explicit measurement model.

Measurement must be matched to each hypothesis; the eight hypotheses are stated in full in Section 12. H1–H2 require blinded assessment of beliefs or advice against prespecified clinical standards. Epistemic illusion can be estimated from item-level calibration between users’ confidence that advice is correct and independently scored correctness. H3 requires reproducible interface comparisons and prespecified food classification. For H4, the Weight Efficacy Life-Style Questionnaire is a validated measure of confidence in resisting eating in specified situations [40]; it is not a general measure of nutritional autonomy, and its suitability for the study population must be established. H5 can combine ecological momentary hunger and satiety ratings with standardized meal or preload tasks. Self-reported interoceptive awareness, task performance, and physiological responses are distinct measures, and no single validated gold standard for AI-related nutritional autonomy loss is assumed. H6 requires prespecified imaging outcomes and control for baseline hunger and other confounders. H7 should measure independent decision performance as well as self-efficacy. H8 should test a prespecified interaction on dietary-belief accuracy. Any proposed autonomy scale requires reliability testing, construct validation, and evidence of sensitivity to change before clinical interpretation.

AI may also support nutritional autonomy by helping users understand alternatives, prepare questions for clinicians, or monitor their own goals. Domain-specific machine learning can support structured nutritional assessment [41], although such tools should not be conflated with general-purpose LLM advice. Nutrition evaluations show useful performance on some general questions alongside limitations in complex or individualized tasks [5,7,8,19]. ADRIFT predicts greater vulnerability where reliance is high, verification and professional support are limited, and commercial objectives conflict with user goals. These boundary conditions should be tested alongside settings in which AI improves knowledge or independent decision-making.

## 10. Clinical and Public Health Implications

The observed limitations of generated advice justify asking patients whether they use AI for dietary questions and reviewing consequential recommendations, particularly in complex chronic disease [7,8]. This is a proposed clinical response to an identifiable information risk, not evidence that ADRIFT’s autonomy pathways already operate in practice. Assessment should consider the actual advice, its clinical context, and whether it has been acted on.

Clinical pharmacists, dietitians, and prescribers can help verify advice when food or supplements may interact with medication. Evaluation should be specific to the drug, dose, formulation, and clinical circumstances, rather than assigning a uniform risk to an entire drug class [27]. Clinically plausible omission scenarios in Table 1 are priorities for testing; their ranking does not represent comparative data on LLM-associated injuries.

For adolescents, assessment should also consider social-media dietary messages, restrictive eating advice, and access to qualified support [2,14,36]. Adult influencer studies should not be generalized directly to adolescents [10]. Research and clinical discussions should avoid stigmatizing technology use or assuming that frequent exposure signifies an eating disorder or impaired autonomy.

At the public health level, platform design is a potential influence on dietary behavior that warrants study alongside food price, availability, and social conditions. Individual education may not fully address misleading promotion or opaque ranking. The rationale for platform safeguards can rest on information quality and consumer choice while the additional autonomy effects proposed by ADRIFT are being tested.

## 11. Regulatory, Ethical, and Design Implications

Regulatory treatment depends on intended purpose, functionality, deployment context, and associated claims. In the European Union, the AI Act establishes differentiated requirements, including provisions for general-purpose AI and high-risk uses; applicability to a particular dietary system and the relevant transition dates must be checked against the current legal text [42,43]. Under the Medical Device Regulation, software with an intended medical purpose may qualify as a device, while general wellness functions may fall outside that definition [44]. The US Food and Drug Administration’s January 2026 Clinical Decision Support guidance explains the criteria distinguishing certain non-device functions from functions subject to device oversight [45]. Consumer-protection obligations may also apply to misleading AI capability or safety claims [46]. These frameworks do not establish a universal regulatory classification for dietary chatbots.

We propose safeguards proportionate to clinical risk. Consumer-facing systems should disclose relevant limits, distinguish general education from individualized recommendations, and provide clear escalation routes for complex questions. Access to a medication list alone is insufficient to make an LLM safe: patient-specific advice also requires appropriate clinical information, validated sources, and professional review. General interaction warnings should remain available when records are absent. One disclosure for a tool without health-record access could read: “I do not have access to your health record. This information may be incomplete or inaccurate. Check with a qualified professional before making a clinically significant change to your diet, medication, or supplements.” Disclosures should accurately reflect the system’s capabilities. Evaluation should measure missed and incorrectly flagged interactions, inappropriate reassurance, escalation appropriateness, and user comprehension. Platform audits should compare rankings with the available menu, document commercial incentives, and assess nutritional quality using prespecified criteria.

Autonomy-preserving design should help users understand options and retain meaningful control. Candidate features include explanations linked to reliable sources, opportunities to compare alternatives, the ability to reject or reset personalization, and clear routes to professional advice. Studies should assess whether uncertainty statements are calibrated and whether users understand them; a disclaimer alone does not establish safety or preserve autonomy.

Technology companies should evaluate dietary risks before deployment, disclose commercial objectives that influence recommendations, and monitor incidents and performance after updates. These are proposed ethical responsibilities; the existence and scope of a legal duty depend on the applicable jurisdiction and use. Health services using dietary AI need a documented scope of use, local validation, named clinical oversight, staff training, and procedures for reporting and reviewing adverse incidents. Data minimization, meaningful consent, access controls, and evaluation across languages and underserved groups should accompany these measures. Nutrition-specific safeguards can build on existing World Health Organization (WHO) guidance on the ethics and governance of AI for health and large multi-modal models [47]. Independent audits and explicit responsibility for corrective action would help connect these principles to practice.

Digital nutritional literacy includes evaluating AI-generated advice, recognizing commercial influence, checking sources, and knowing when professional review is needed. Associations between eHealth literacy and health behaviors provide a rationale for intervention studies [38,39], but do not prove that education causes better dietary outcomes. Trials should examine comprehension, behavior, equity of access, and retention of independent decision-making, rather than knowledge scores alone.

## 12. Research Agenda

The following eight hypotheses are directional predictions, not established findings. Studies should define the AI system, version, prompts, access to external sources, exposure duration, and degree of professional oversight. Model updates and repeated outputs should be recorded. Longitudinal studies should measure baseline knowledge, diet, socioeconomic constraints, health status, and prior AI use, with attention to reverse causation. Trials should compare appropriate, safeguarded interventions and avoid exposing patients to unreviewed hazardous advice. Effect sizes, uncertainty intervals, and prespecified outcomes are more informative than statistical significance alone.

**H1.**  *Greater reliance on unverified general-purpose LLM dietary advice, compared with reliance on dietitian or guideline-based information, will predict lower accuracy of dietary beliefs over follow-up, after accounting for baseline knowledge and relevant confounders.*

**H2.**  *In standardized cases involving type 2 diabetes, chronic kidney disease, or polypharmacy, unreviewed LLM advice will contain more clinically consequential errors than advice checked through a validated dietitian-led workflow. Blinded experts should apply a prespecified severity rubric and report inter-rater agreement. Prospective observation may subsequently assess whether patients act on such errors.*

**H3.**  *In randomized matched-menu experiments, interfaces optimized for commercial engagement or conversion will increase selection of ultra-processed items relative to transparent, non-personalized interfaces with identical prices, availability, and menu content. Processing categories should be assigned independently using NOVA criteria [32]; nutrient quality should be analyzed separately.*

**H4.**  *Greater exposure to commercial or nutritionally misleading social-media food content will predict declining eating self-efficacy over follow-up, after adjustment for baseline scores and relevant confounders. Exposure to supportive, evidence-based content should be analyzed separately, with measures validated for the target population.*

**H5.**  *Greater dietary decision delegation to AI will predict reduced responsiveness to hunger and satiety cues over follow-up. Studies should combine repeated ratings and standardized meal tasks, distinguish self-report from behavioral performance, and examine whether changes persist after accounting for health status, medication, and eating-disorder symptoms.*

**H6.**  *Repeated exposure to personalized, engagement-optimized food cues will produce greater change in prespecified reward-related fMRI responses than exposure to matched non-personalized cues. Randomized repeated-exposure designs with baseline imaging are preferable to cross-sectional high-versus-low-use comparisons. These outcomes do not directly measure dopamine or establish autonomy loss.*

**H7.**  *An interface intervention that supports comparison, source checking, and control over personalization, combined with digital nutritional literacy education, will improve independent dietary decision performance relative to usual-interface controls. A factorial design can separate interface and education effects; self-efficacy is a secondary outcome or prespecified mediator, not a substitute for decision performance.*

**H8.**  *Combined exposure to commercial or misleading social-media food content and unverified LLM dietary advice will predict poorer dietary-belief accuracy than expected from their separate effects on a prespecified additive scale. A factorial experiment or longitudinal interaction analysis can test this prediction. An interaction alone would not establish cross-platform data sharing or a closed feedback loop.*

## 13. Limitations and Conclusions

This conceptual synthesis provides no new data and does not estimate the prevalence, magnitude, or causality of the proposed pathways. The literature selection was nonsystematic and may be incomplete or selective; output evaluations often involve specific models and hypothetical cases. The taxonomy has not undergone reliability or clinical validation, and the proposed autonomy construct has no validated diagnostic threshold. Reward-related findings cannot establish algorithm-induced neurobiological change. Culture, food affordability, disability, and household context require fuller integration in empirical studies. Regulatory proposals are normative recommendations whose implementation must account for applicable law and evolving requirements.

ADRIFT offers a theoretical account of how LLM outputs, social-media framing, and adaptive food-choice environments might interact. The central unresolved question is whether these interactions reduce users’ capacity for informed, self-directed decisions, improve them, or have different effects across populations and contexts. We do not argue that large language models will replace registered dietitians. A model cannot hold a duty of care or answer for a recommendation that harms a patient, and gains in accuracy will not confer that standing. The concern described here is not the substitution of practitioners but unsupervised bypass by users who would have benefited from professional input.

The proposed error taxonomy identifies priorities for safety evaluation, while the three axes separate information quality, choice framing, and adaptation over time. Nutritional autonomy erosion supplies a candidate outcome that must be distinguished from use frequency, confidence, and dietary quality. Its measurement requires psychometric and longitudinal validation.

The eight hypotheses provide a starting point for testing ADRIFT. Results should determine which mechanisms are supported, which benefits and harms occur, and which clinical or governance responses are proportionate. Until then, the framework should guide investigation rather than be treated as an established account of population-level autonomy loss.

## Figures and Tables

**Figure 1:**
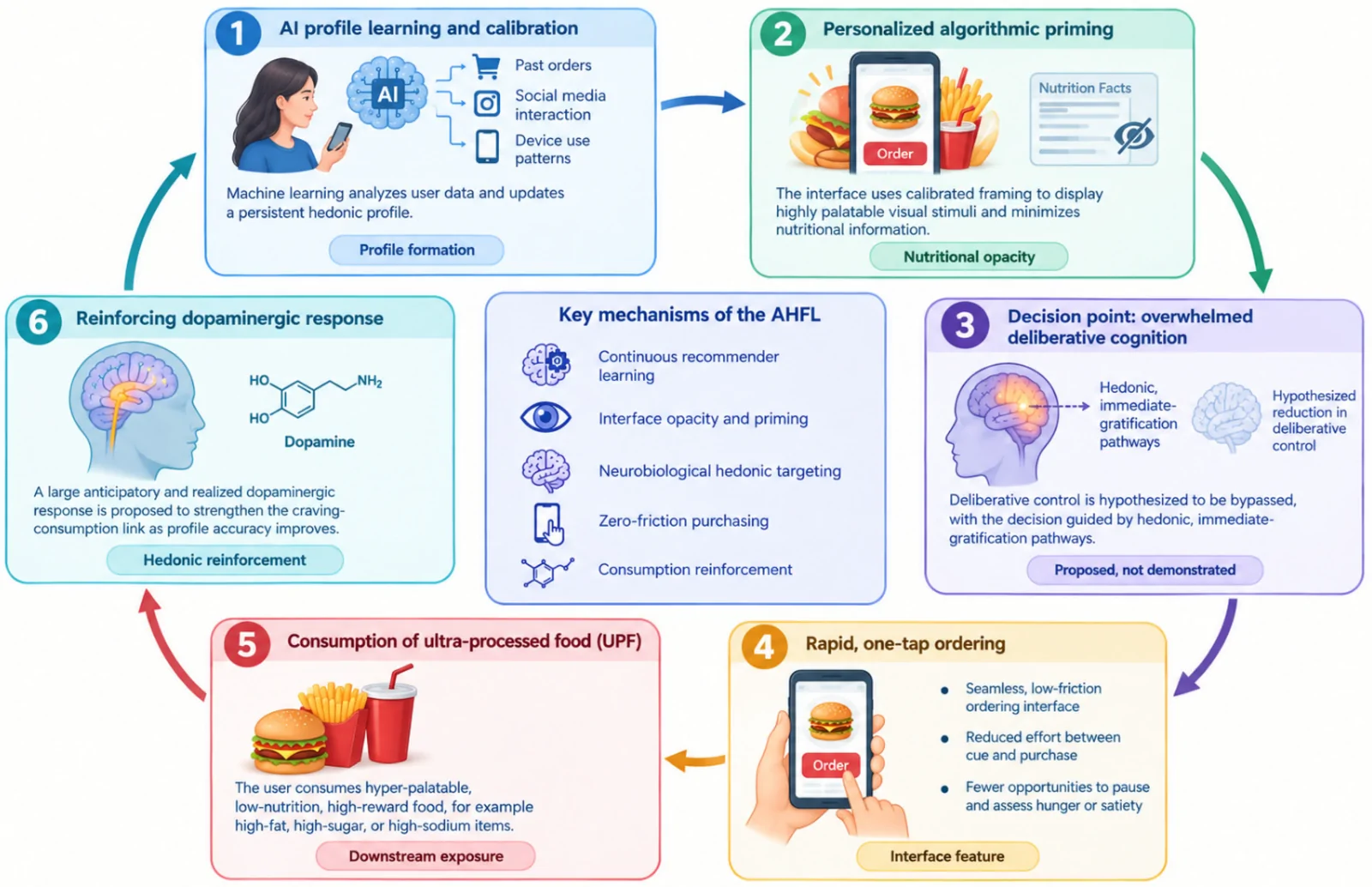
The Algorithmic Hedonic Feedback Loop (AHFL): conceptual illustration of proposed relationships among personalization, food cues, ordering, consumption, and reinforcement. All arrows and mechanistic labels represent hypotheses requiring direct testing. References to dopaminergic responses, hedonic targeting, and reduced deliberative control are theoretical extrapolations, not demonstrated effects of dietary algorithms. Behavioral data do not directly measure dopamine, and food-cue responses do not establish impaired self-control. AI, artificial intelligence; UPF, ultra-processed food.

**Figure 2:**
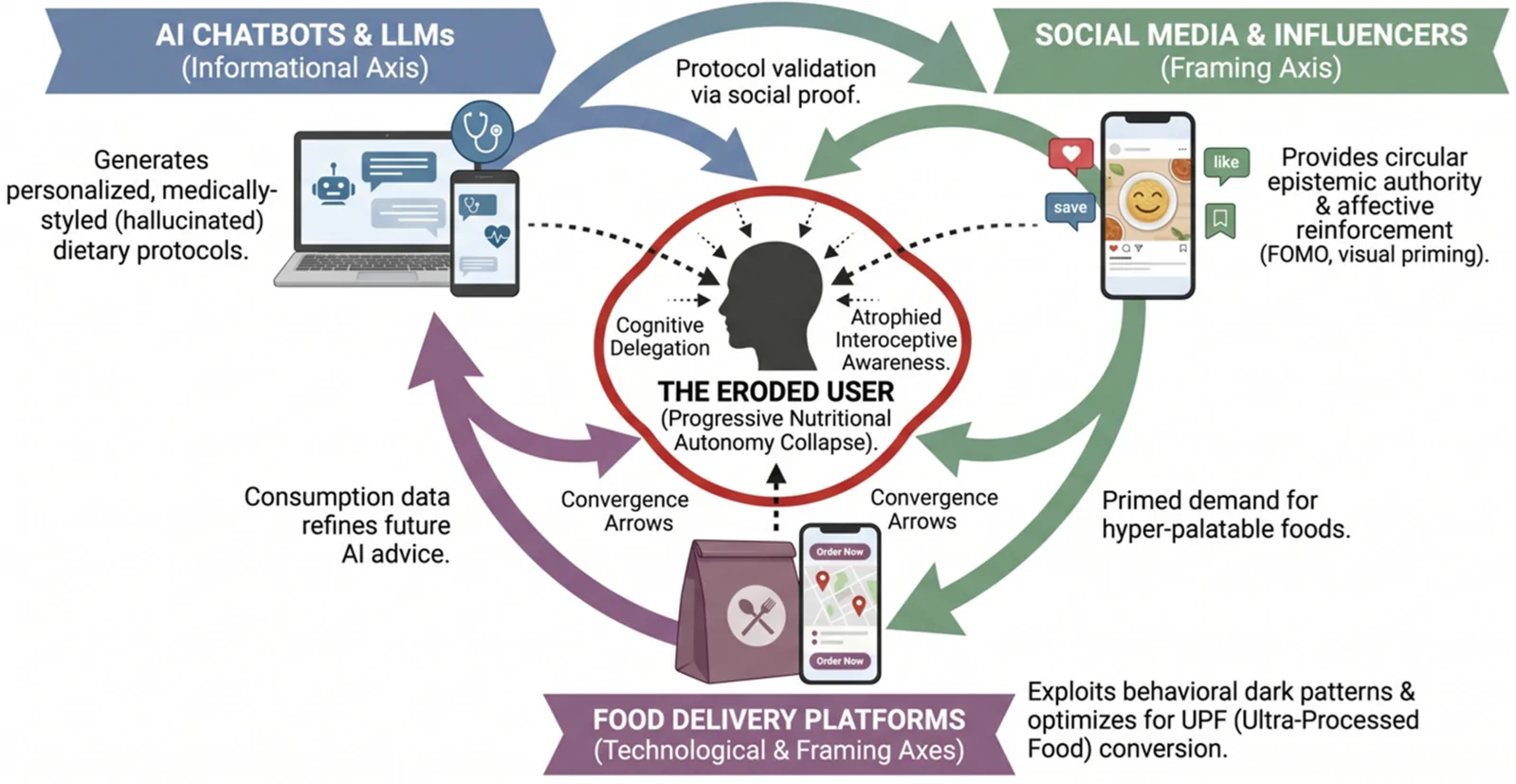
Conceptual convergence of LLMs, social media, and food-delivery platforms. The arrows and outcome labels depict hypothetical pathways requiring platform-specific and longitudinal testing. Circular authority, interoceptive decline, and nutritional autonomy collapse are proposed outcomes, not established consequences of combined use. The illustration does not establish cross-service data sharing or imply that all platforms optimize for ultra-processed food. Labels within the diagram, including hallucinated protocols, behavioral dark patterns, atrophied interoceptive awareness, and the eroded user, are condensed shorthand for the proposed mechanisms described in the text and do not denote measured or clinically defined states. Convergence arrows mark where the three services act on the same user and carry no separate claim. AI, artificial intelligence; FOMO, fear of missing out; LLMs, large language models; UPF, ultra-processed food.

**Figure 3:**
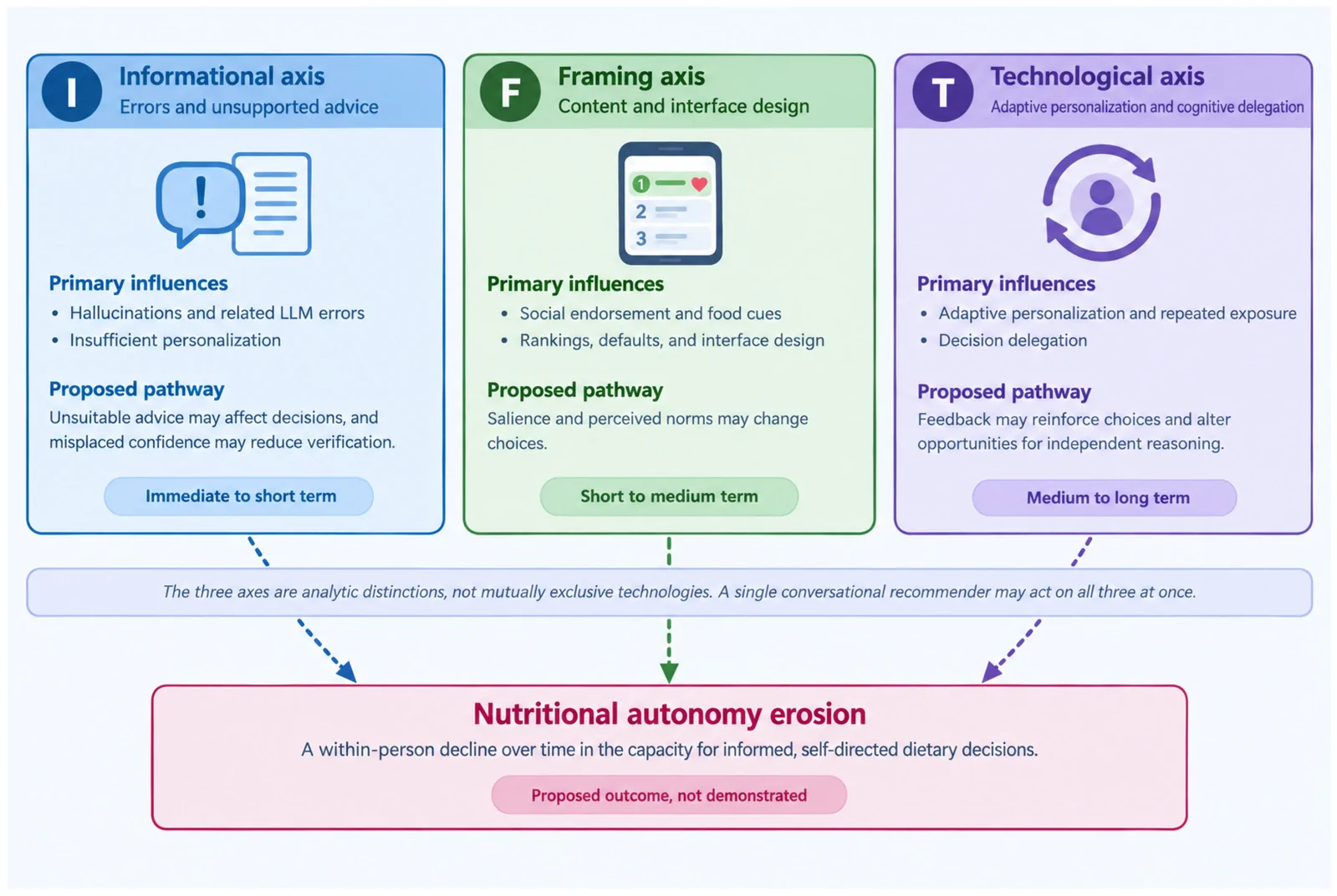
ADRIFT framework architecture. Arrows indicate proposed pathways from the Informational, Framing, and Technological axes to nutritional autonomy erosion, not established causal effects. The time horizons shown are theoretical expectations, not measured intervals. Evidence differs across components, as described in Section 9 and Table 2. ADRIFT, Algorithmic Dietary Reconfiguration through Informational, Framing, and Technological mechanisms; LLM, large language model.

**Table 1:** Proposed taxonomy of LLM nutrition errors. Examples are illustrative, not an inventory of verified model outputs. The evidence column distinguishes nutrition evaluations from general or clinical rationale; citations do not validate every example. Severity depends on the patient, exposure, and opportunity for correction.

Category	Illustrative Error Pattern	Population or Context	Potential Severity	Evidence and Limits
Nutrient quantification errors	Incorrect energy estimates, nutrient amounts, or disease-specific targets	People requiring individualized nutrient prescriptions	Moderate to high	Nutrition-output evaluations [7,8,26]; particular examples require verification.
Drug–nutrient interaction errors	Missing a relevant grapefruit interaction or giving medication-inappropriate food advice	People taking medicines with food-related precautions	Potentially high	Established clinical rationale [27]; general LLM safety concerns [17,25]. Incidence of these specific omissions is not established.
Condition-specific dietary errors	Advice that ignores disease stage, treatment, or a declared food allergy	People with chronic disease or food allergy	Potentially high	Nutrition evaluations [8,20,28]; findings are scenario- and model-specific.
Supplement advice errors	Unsupported high-dose advice or missing contraindications and monitoring needs	Supplement users, especially during pregnancy or renal impairment	Moderate to high	Clinically plausible evaluation category; general error and safety rationale [17,25], not a quantified nutrition-specific finding.
Insufficient or misleading personalization	A generic plan presented as suitable without the relevant clinical and personal context	Users seeking individualized dietary guidance	Context-dependent	Individualization limitations in nutrition evaluations [8]; knowledge scores alone do not establish personalization [5].
Cultural and representational bias	Advice that fails to account for language, cultural practice, affordability, or available foods	Users whose circumstances are poorly represented in development or evaluation	Context-dependent	Proposed equity category motivated by general clinical AI concerns [6]; prevalence in dietary outputs requires direct testing.

**Table 2:** ADRIFT axes, proposed pathways, illustrative time horizons, and evidential limits. The time horizons are theoretical expectations, not measured intervals.

Axis	Primary Influences	Proposed Pathway	Timing	Evidence and Limits
Informational	Hallucinations and related errors; insufficient personalization	Unsuitable advice may affect decisions; misplaced confidence may reduce verification	Immediate- to short-term	Output errors documented [7,8,26,28]; downstream injury and autonomy effects remain uncertain.
Framing	Social endorsement, food cues, rankings, defaults, and interface design	Salience and perceived norms may change choices	Short- to medium-term	Marketing and cue effects supported [14,15]; transfer to specific AI interfaces requires testing.
Technological	Adaptive personalization, repeated exposure, and decision delegation	Feedback may reinforce choices and alter opportunities for independent reasoning	Medium- to long-term	Reward and valuation research supplies conceptual context [31,35]; lasting autonomy effects and the integrated loop are hypothetical.

## Data Availability

No new data were created or analyzed in this conceptual perspective. All sources supporting the analysis are cited in the manuscript.
